# DoSGuard: Mitigating Denial-of-Service Attacks in Software-Defined Networks

**DOI:** 10.3390/s22031061

**Published:** 2022-01-29

**Authors:** Jishuai Li, Tengfei Tu, Yongsheng Li, Sujuan Qin, Yijie Shi, Qiaoyan Wen

**Affiliations:** State Key Laboratory of Networking and Switching Technology, Beijing University of Posts and Telecommunications, Beijing 100876, China; sky_lee1990@bupt.edu.cn (J.L.); tutengfei.kevin@bupt.edu.cn (T.T.); lee_yongsheng@bupt.edu.cn (Y.L.); yijieshi2000@bupt.edu.cn (Y.S.); wqy@bupt.edu.cn (Q.W.)

**Keywords:** software-defined networking (SDN), denial-of-service (DoS), fake source address, OpenFlow, anomaly detection, defense

## Abstract

Software-defined networking (SDN) is a new networking paradigm that realizes the fast management and optimal configuration of network resources by decoupling control logic and forwarding functions. However, centralized network architecture brings new security problems, and denial-of-service (DoS) attacks are among the most critical threats. Due to the lack of an effective message-verification mechanism in SDN, attackers can easily launch a DoS attack by faking the source address information. This paper presents DoSGuard, an efficient and protocol-independent defense framework for SDN networks to detect and mitigate such attacks. DoSGuard is a lightweight extension module on SDN controllers that mainly consists of three key components: a monitor, a detector, and a mitigator. The monitor maintains the information between the switches and the hosts for anomaly detection. The detector utilizes OpenFlow message and flow features to detect the attack. The mitigator protects networks by filtering malicious packets. We implement a prototype of DoSGuard in the floodlight controller and evaluate its effectiveness in a simulation environment. Experimental results show the DoSGuard achieves 98.72% detecion precision, and the average CPU utilization of the controller is only around 8%. The results demonstrate that DoSGuard can effectively mitigate DoS attacks against SDN with limited overhead.

## 1. Introduction

Software-defined networking (SDN) is a new network technology and architecture. It has been widely recognized by academia and industry and has been successfully applied to various fields, such as enterprise networks and data centers [1]. However, the idea of the separation of logical control and forwarding functions expands the attack surface, and the control plane, data plane, and application plane will face security challenges [2,3]. The denial-of-service (DoS) attack is one of the most severe network security threats. In SDN, the data plane is responsible for data processing and packet forwarding. When the unmatched packets are received, the switch will send them to the controller via *Packet-In* messages. The controller processes *Packet-In* messages from the switch and sends flow rules to the switch in the form of *Flow-Mod* messages [4]. Since the SDN switch sends all packets with unknown flows to the controller [5], a DoS attacker can easily exploit this fact and send a stream of unmatched flows. Due to the massive amount of spoofed flow requests, the controller’s processing capabilities will be overloaded and cannot respond quickly to the legitimate user. At the same time, the controller must install many flow entries for each spoofed flow. The flow table of the switches with scarce resources can easily overflow. Moreover, these unmatched flows would consume the controller CPU, the bandwidth between the data plane and control plane, and the switch’s CPU resources. These limited resources would lead to new DoS threats [6].

Some existing works try to address SDN DoS attacks, but they have their limitations. SLMU [7] can determine whether an attack occurs by collecting necessary statistical information. However, it needs to be installed on every switch and increases deployment and management costs. PacketChecker [8] only defends against packet-injection attacks based on a MAC address. The INSPECTOR [9] protects a compromised controller by verifying *Packet-In* messages, tops the attack efficiently, and enhances the performance of the controller under malicious attack. As a hardware device added to the SDN architecture, INSPECTOR requires additional measures to ensure that it is not damaged. Tian et al. [10] proposed an attack-detection method based on conditional entropy. This solution can effectively detect whether an attack occurs, but it cannot mitigate and inhibit the occurrence of the attack. To defend against SDN-aimed DoS attacks from the source, the authors of [6] proposed DosDefender and realized attack-detection by verifying the legality of the *Packet-In* messages. However, this solution uses a threshold-based approach to detect attacks that falsify source ports, which can easily lead to false positives. All of the above works have obvious shortcomings. As a comparison, we summarize different approaches based on various drawbacks, as shown in Table 1.

This paper focuses on the detection and defense methods of DoS attacks implemented by forging source address information in SDN. To complete this work, we face the following two main challenges that need to be resolved:

- How to respond to network abnormalities on time, that is, how to quickly find abnormal hosts in the network;
- How to precisely detect SDN-aimed attacks and effectively defend against them.

For the first challenge, motivated by existing work [6], we have achieved the consistency verification of *Packet-In* messages by maintaining the mapping relationship between the switch and the host connected to this switch. If the host’s validation fails, we think it is abnormal. For the second challenge, when an abnormal host is found, we will use the message rate and flow frequency features to detect attacks, thereby reducing the false alerts effectively. Simultaneously, once an attack is detected, we install flow rules to the switch that connects these hosts to drop the malicious host’s traffic. We propose DoSGuard, a scalable and protocol-independent defense system in OpenFlow networks, to implement the scheme described above. DoSGuard has three components: a monitor, a detector, and a mitigator. The monitor verifies the consistency of *Packet-In* messages and finds abnormal hosts in the network. The detector uses features such as message rate and flow frequency to detect whether an attack occurs. The mitigator is responsible for installing blocking rules against malicious hosts. These components cooperate to protect the SDN network effectively.

In general, our paper makes the following contributions:

- We propose an attack-detection mechanism that consists of anomaly detection by maintaining information between the switches and the hosts, and attack-detection based on OpenFlow message and flow features, effectively reducing the false-alerts attack in the SDN environment;
- We design and implement DoSGuard by extending the SDN controller to prevent these SDN-aimed DoS attacks. This scheme does not require additional hardware equipment or any data-plane modifications;
- We evaluate the effectiveness of DoSGuard in OpenFlow software environments. The results demonstrate that DoSGuard is effective with limited overhead.

The rest of the paper is organized as follows. In Section 2, we present the security problem and related work. We detail the implementation in Section 3 and performance evaluation in Section 4. In Section 5, we discuss the advantages and limitations of DoSGuard and, finally, conclude our work in Section 6.

## 2. SDN Security and Related Works

### 2.1. SDN Security

**Figure 1:**
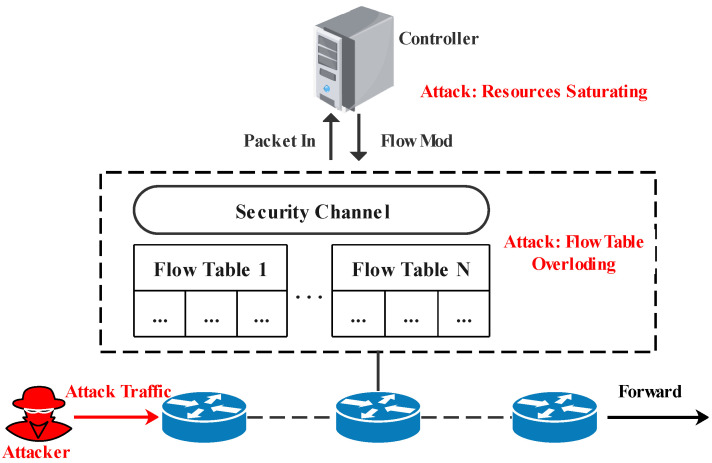
An example of a DoS attack [11].

A centralized network architecture makes the controller the main target of DoS attacks. Figure 1 shows an example of a DoS attack by forging the source address. As shown in the figure, each switch is composed of one or more flow tables. When the first packet of a new flow is recieved, the switch looks up the flow table to determine how to process this packet. If there is a flow entry that matches the packet, the associated action is executed. In contrast, if no match is found, the switch encapsulates the packet into a *Packet-In* message and sends it to the controller over the secure channel. The controller determines how to process the new packet and installs new rules into the switch by generating *Flow-Mod* messages. As a consequence, the switch processes the current packet according to the specified instructions. Therefore, if an attacker injects enormous spoofed traffic into the switch in a short period, it will seriously consume the computing resources of the controller. Due to frequent decision-making requests, the controller cannot respond to legitimate users on time, affecting network quality. Simultaneously, the switch needs to install flow entries, which easily increase the flow table’s space occupation and eventually causes the flow table to be overloaded.

### 2.2. Related Works

Due to the attributes of centralized control and programmability, SDN can provide more advanced network monitoring, flow control, and security analysis. Therefore, early research focused mainly on using SDN to empower traditional networks. However, with the widespread application of SDN, its security problems have become more and more prominent. The security research of SDN has gradually become a new hot spot, especially for the security research of the data plane [12,13,14,15,16,17,18,19] and control plane [6,11,20,21].

For the security research of the data plane, SPHINX [12] detected both known and potentially unknown attacks on network topology and data-plane forwarding originating within an SDN by leveraging the novel abstraction of flow graphs. However, the attacker can bypass this method by distributing or slowing down the attack procedure [9]. FTGuard [13] implemented a behavior-based priority-aware defense strategy to cope with the flow table overflow attack. It differentiates flows based on the priority assigned to each user, with flows from benign users likely to receive high scores and have higher priority. In comparison, flows from suspicious users have lower priority. However, the user ratings are at risk of contamination. WedgeTail [14] distinguishes malicious forwarding devices by computing the expected and actual trajectories of packets, effectively protecting the data plane. This approach is useful, but deploying it in a real-world network is challenging. In [15], the authors proposed a machine-learning approach to detect DoS attacks on SDN data-plane switches using flow table information and OpenFlow traffic. In addition, they also evaluated three different algorithms, namely, neural networks, support vector machines, and naive Bayes classifiers. However, the detection mechanism follows a static approach, and how working it in real-time is still a problem that needs to be solved. The authors of [16] proposed a flow table sharing mechanism which effectively mitigates the damage to the normal network caused by the flow table overloading attack. This method only mitigates but cannot detect the occurrence of an attack. CCD [17] analyzes the rules correlation based on packet header fields and resolved any identified rule conflicts in real time before rule installation to prevent covert channel attacks effectively. However, additional mechanisms are required to verify the reachability of the header change rules. In [18], the authors proposed a QoS-aware mitigation strategy, which integrated the available idle flow table resource of the whole SDN system to mitigate overloading attacks on a single switch of the system. However, the timeout operation can lead to legitimate flows being denied entry into the network. vSwitchGuard [19] aims to identify the victim switches targeted by known or unknown types of saturation attacks with machine-learning classifiers. However, the paper only studied five saturation attacks in the SDN environment, and more types of attacks need to be investigated.

For the security research of the control plane, the authors of [20] performed a security analysis of OpenFlow using STRIDE and attack-tree modeling methods. They further proposed appropriate countermeasures to mitigate these security issues. In [22], the authors described several threat vectors that may enable the exploitation of SDN vulnerabilities and then sketched the design of a secure and dependable SDN control platform. The authors of [4] proposed new SDN-specific attack vectors which can effectively poison the network topology. To defend against these attacks, they designed TopoGuard, which provides automatic and real-time detection of network topology exploitations. However, it does not consider the origin of *Packet-In* messages in solving the host location hijacking attack. The authors of [23] studied the fingerprinting of controller–switch interactions by a remote adversary concerning various network parameters. They presented and evaluated an efficient countermeasure to strengthen SDN networks against fingerprinting. Since the centralized network architecture is more likely to be the target of DoS attacks, more research focuses on the detection and defense of DoS attacks in SDN. In [24], the authors built a mechanism that used statistical data to monitor the network and differentiate DoS traffic from benign traffic using entropy in an SDN environment. However, statistical solutions in SDN architectures display some flaws that need to be addressed to realize efficient methods for detecting and mitigating these security risks. Neural networks were used to detect DoS attacks in [25,26]. An online defense system for SDN network environments against DDoS and port scan attacks was proposed in [25]. In [26], an accurate DDoS detection method using the lion optimization algorithm, combined with CNN, is proposed. SDN-Guard [27] mitigates DoS attacks in SDN by dynamically managing flow rules. Although it performed well in defending against DoS attacks, additional hardware equipment was required. FloodDefender [21] improves the flow table utilization, time delay, and packet loss rate based on three novel techniques. SDNManager [28] was proposed as a lightweight and fast detection and mitigation system. However, it has a performance penalty. In addition, BWManager [11] implements a novel SDN controller-scheduling algorithm based on bandwidth prediction. The evaluation results show BWManager provided the QoS with a limited overhead in both hardware and software environments. As an extension module of the SDN controller, DoSDedender [6] defends against DoS attacks by maintaining the mapping relationship between the host and the switch. This solution uses a threshold-based approach to detect attacks that falsify source ports, which can easily lead to false positives.

This paper focuses on the detection and defense methods of DoS attacks implemented by forging source address information in SDN. By comparing existing works, we can see that designing a defense system that integrates fast anomaly discovery and accurate attack detection is necessary. For this purpose, we propose DoSGurd, and will introduce it in detail in the next section.

## 3. System Overview

In this section, we briefly introduce DoSGuard and describe each component of DoSGuard in detail.

### 3.1. System Architecture

**Figure 2:**
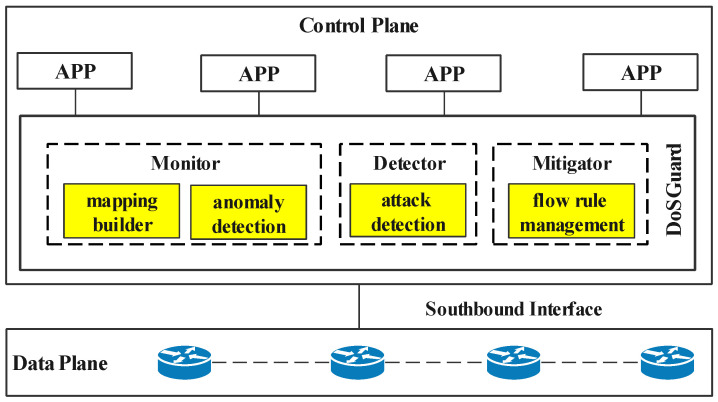
Architecture of DoSGuard.

DoSGuard stands between the controller platform and other controller apps, as depicted in Figure 2. It consists of three core components: a monitor, a detector, and a mitigator. The monitor realizes the rapid discovery of network anomalies by constructing the mapping relationship between the switches and the hosts. The detector utilizes OpenFlow message and flow features to detect the attack. The mitigator is responsible for defense against attacks by filtering malicious packets. These various components cooperate to complete anomaly findings, attack detection, and attack defense.

**Figure 3:**
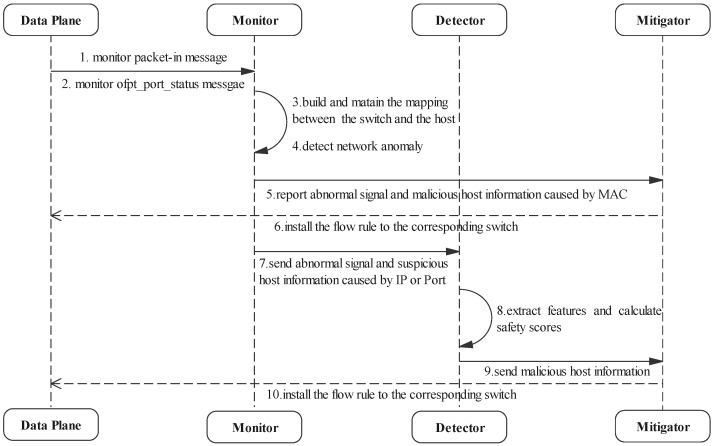
Workflow of DoSGuard.

For better understanding, we use Table 2 to show the meanings of major notations. In addition, in order to facilitate analysis, we show the workflow of DoSGuard in Figure 3. Next, we will introduce the functions of each component in detail.

### 3.2. Monitor

The monitor realizes the rapid discovery of network anomalies by constructing the mapping relationship between the switches and the hosts inspired by DoSDefender [6]. On the one hand, it listens to *Packet-In* messages from the data plane and extracts relevant switch and host information. The switch information includes the datapath ID and the ingress port, where the packets come from. The host information includes the MAC address, the IP address, and the port. On the other hand, it monitors the OFPT_PORT_STATUS message from the data plane to realize the dynamic management of the mapping information between the switch and the host. By tracking the mapping information changes between the switch and the host in real time, DoSGuard can quickly find network anomalies.

The monitor consists of two components: mapping builder and anomaly detection. The mapping builder is responsible for constructing and maintaining the mapping relationship between switches and hosts. The anomaly detection is responsible for the rapid discovery of network anomalies.

#### 3.2.1. Mapping Builder

The mapping builder is responsible for constructing the mapping relationship between the switch and the host. For ease of presentation, we assume that each switch is connected to only one host at most. On this assumption, we give the following definition.

We consider a set of switches $S=\{s_{1},s_{2},\ldots ,s_{n}\}$ involved in the data plane, where *n* is the number of switches. For each $s_{j}$ in *S*, it is connected to, at most, + one host.

We consider a set of hosts $H=\{h_{i}=\left(mac_{i},ip_{i},ports_{i}\right)|i=1,2,\ldots ,m\}$, where *m* is the number of hosts. Each $h_{i}$ in *H* includes $mac_{i}$, $ip_{i}$, and $ports_{i}$. The $mac_{i}$ represents MAC address, the $ip_{i}$ denotes IP address, and the $ports_{i}$ is a set of port numbers that $h_{i}$ uses.

Let $T=\{<s_{j},h_{i}>|j<=n,i<=m\}$ denote a mapping table for the relationship between switches *S* and hosts *H*. Each entry in *T* consists of the $s_{j}$ and $h_{i}$, which means $s_{j}$ and $h_{i}$ are connected. Moreover, we define a variable $X_{j}$ to denote whether *T* contains a mapping entry associated with $s_{j}$. For example, $X_{1}=1$ means an entry in *T* consists of $s_{1}$ and the host connected to $s_{1}$.

(1) $$X_{j}=\left\{\begin{array}{cc} 1 & \mathrm{entry}\;\mathrm{related}\;\mathrm{to}\;s_{j}\;\mathrm{in}\;T\\ 0 & \mathrm{entry}\;\mathrm{related}\;\mathrm{to}\;s_{j}\;\mathrm{not}\;\mathrm{in}\;T \end{array}\right.$$

The mapping builder module listens for the data plane messages and maintains the mapping table. When a *Packet-In* message is received, it will extract the included switch and host information, such as datapath ID, ingress port, MAC address, IP address, and port number. The mapping builder process is shown in Algorithm 1, which mainly includes three stages:

- **Monitor message (line 1–3)**: The mapping builder module creates a mapping relationship table T and listens for messages from switch $s_{j}$ on the data plane.
- **Build mapping (line 4–9)**: When the mapping builder module receives a *Packet-In* message, it extracts the MAC address, IP address, and port number of the source host and looks for *T*. If *T* contains a mapping entry of $s_{j}$ (i.e., $X_{j}=1$), it adds a port number to the port set of $h_{i}$ associated with $s_{j}$; otherwise, it adds the mapping of $s_{j}$ and $h_{i}$ to *T*.
- **Remove mapping (line 10–11)**: The mapping builder module removes the entry related to $s_{j}$ in *T* when it receives an OFPT_PORT_STATUS message. In this way, it maintains information in a dynamic and real-time manner.

**Algorithm 1** Mechanism of Mapping Builder.**Input:** *S*: set of switches, *H*: set of hosts**Output:***T*: mapping table of switches and hosts  1:Initialize *T* = *⌀*;  2:**for**$\forall s_{j}\in S$**do**  3:    Listening message from $s_{j}$  4:    **if** The message is *Packet-In*, **then**  5:        Extract MAC, IP, port of source host  6:        **if** $X_{j}=0$, **then**  7:           $h_{i}$⟵ (MAC, IP, ⌀)  8:           *T*⟵$<s_{j},h_{i}>$  9:        Add port to $ports_{i}$ related to $s_{j}$ in *T*10:    **if** The message is *ofp_port_status*, **then**11:        Remove entry related to $s_{j}$ from *T*12:    **return** *T*

#### 3.2.2. Anomaly Detection

The anomaly-detection module tracks the changes of the mapping information between the switch and the host in real time. By verifying the consistency of information, it can quickly find the network abnormality. In this paper, we define three types of anomalies according to different inducements, and the detailed description and judgment methods are as follows.

(1) Anomaly caused by inconsistent MAC (AIM)

When the anomaly-detection module receives a *Packet-In* message from $s_{j}$, it considers it an abnormity if the extracted source MAC address differs from the MAC address of $h_{i}$ associated with $s_{j}$ in *T*.

(2) Anomaly caused by inconsistent IP (AII)

When the anomaly-detection module receives a *Packet-In* message from $s_{j}$, it considers it an abnormity if the extracted source IP address differs from the MAC address of $h_{i}$-associated with $s_{j}$ in *T*.

(3) Anomaly caused by inconsistent Port (AIP)

Under normal conditions, the number of host ports grows steadily. When a DoS attack occurs, the attacker usually randomly selects ports for flows, and the value of port growth rate (PGR) sharply increases. The equation of PGR is shown below:

(2) $$PGR=\frac{PortNum}{interval},$$

where PortNum is the number of active ports and interval represents the time interval.

The anomaly-detection module distinguishes AIP based on PGR. It judges an abnormity if $PGR_{i}$ (the port growth rate of $h_{i}$) is greater than the threshold δ within the time interval *t*.

The anomaly-detection process is shown in Algorithm 2. It monitors *Packet-In* messages from $s_{j}$ and extracts source MAC addresses and IP addresses. If the MAC address or IP address does not match the information of the host $h_{i}$ associated with $s_{j}$ in *T*, it judges the host as suspicious. At the same time, it periodically calculates the $PGR_{i}$ of $h_{i}$. If $PGR_{i}$ is greater than the threshold δ, it considers it as an abnormality; otherwise, it will clear $ports_{i}$ of $h_{i}$. It is worth noting that, unlike handling AII or AIP, the anomaly-detection module notifies the mitigator to block for AIM, rather than the detector module. The main reasons are as follows:

- The host’s MAC address of a physical device is usually unchanged in realistic networks [6];
- An OFPT_PORT_STATUS message is triggered to notify the controller when the host location is migrated (such as leaving or joining the network);
- The attack through forging a MAC address will have a fatal impact on the SDN topology.

**Algorithm 2** Mechanism of Anomaly Detection.  1:**for**$\forall s_{j}\in S$, **do**  2:    Listening *Packet-In* message from $s_{j}$  3:    Extract MAC, IP of source host  4:    **if** $mac_{i}$≠ MAC, **then**  5:        Report AIM to mitigator module  6:    **if** $ip_{i}$≠ IP, **then**  7:        Report AII to detector module  8:**for**$\forall h_{i}\in T$, **do**  9:    Caculate $PGR_{i}=\frac{ports_{i}.size}{t}$10:    **if** $PGR_{i}\geq \delta$, **then**11:        Report to detector module12:    **else**13:        Remove all port from $ports_{i}$

In summary, we will directly install blocking rules through mitigator to protect SDN when AIM occurs.

### 3.3. Detector

The detector periodically collects the message and flow information of switches. When the detector module receives an abnormal signal from the anomaly detection module, it extracts suspicious switch and host information for attack detection. It will calculate a security score based on the features collected within the interval time. Once the attack is confirmed, it informs the mitigator to block immediately.

Although utilizing CPU, memory, and bandwidth can effectively identify potential attacks, this detection may lead to false alerts in large networks [29]. Some studies assume that an attack has occurred when information is inconsistent, and then adopt blocking strategies [6]. It is also easy to misjudge an attack by forging a source IP or port. To accurately identify attacks, we comprehensively consider the characteristics and effects of DoS attacks and use them to make decisions. The extracted features are: (1) rate of *Packet-In* messages, (2) rate of flow rules, (3) average packet number of flows, (4) average duration time of flows, (5) entropy of the source IP addresses, and (6) growth rate of source ports.

(1) Rate of *Packet-In* messages (RPM)

An attacker carries out DoS attacks by sending a large number of spoofed flows towards the switch in a short period. As a consequence, the switch frequently requests the controller via *Packet-In* messages, which makes the message rate increase significantly. Therefore, we take the *Packet-In* message rate as an essential parameter to identify the occurrence of an attack and use the following formula for calculation:

(3) $$RPM=\frac{PMNum}{interval},$$

where PMNum denotes the total number of *Packet-In* messages, and interval is the data sampling period.

(2) Rate of flow rules (RFE)

When an attacker launches a DoS attack, the controller must generate some flow entries to establish a route for each spoofed flow. Thus, the number of flow rules related to the malicious host increases sharply. The equation of RFE is as follows:

(4) $$RFE=\frac{FENum}{interval},$$

where FENum represents the total number of flow entries.

(3) Average packet number of flows (APF)

A DoS attack can continuously generate massive flows in a short time, and the number of packets in each flow is minor (e.g., about 1∼3 packets per flow) [11]. Therefore, the average number of packets per flow can be used to evaluate the severity of the attack. The equation of APF is as follows:

(5) $$APF=\frac{\sum_{i}^{FlowNum}PacketsNum_{i}}{FlowNum},$$

where $PacketsNum_{i}$ is the packet number of the *i*th flow and FlowNum is the total number of flows.

(4) Average duration time of flows (ADF)

Since most attack packets with the same information appear only once, corresponding flow rules installed by the controller will not stay for a long time before timeout. When a DoS attack occurs, massive invalid packets make the average duration of each flow reduce sharply. The equation of ADF is shown below:

(6) $$ADF=\frac{\sum_{i}^{FlowNum}Duration_{i}}{FlowNum},$$

where $Duration_{i}$ denotes the time duration of the flow rule for the $i_{th}$ flow.

(5) Entropy of source IP addresses (ESIA)

The source IP addresses distribute randomness when forged by an attacker to carry out a DoS attack, and the entropy increases significantly compared to benign traffic. Therefore, we utilize the entropy of source IP addresses to distinguish network status. The equation of ESIA is as follows:

(7) $$ESIA=-\sum_{i}^{n}\frac{FlowIP_{ij}}{FlowIP_{j}}\log_{}\frac{FlowIP_{ij}}{FlowIP_{j}},$$

where $FlowIP_{i}$ represents the number of flow entries to the $IP_{j}$, and $FlowIP_{ij}$ is the number of flow entries from $IP_{i}$ to the $IP_{j}$.

(6) Growth rate of source ports (PGR)

Since the attacker usually randomly selects ports for flows during a DoS attack, the host port usage sharply increases compared to a benign network. Therefore, the growth of source ports can be used to judge network status. We compute PGR in Formula (2).

The detector module implements a detection scheme based on machine learning (ML) using the above six-tuple feature. At present, ML has become an effective technology for providing network security. Unlike traditional solutions, ML enables the network to identify attacks automatically. It can not only detect known attacks but also identify unknown threats. At the same time, considering the number of features, complexity, robustness, etc., we use support vector machine (SVM) as the classifier. SVM is a supervised learning method used for classification, regression, and outliers detection. This classification algorithm is robust even with noisy training data. The algorithm evaluation experiment is introduced in Section 4. When an attack is detected, the detector module notifies the mitigator to block the malicious host. Simultaneously, the attack detection result will be synchronously fed back to the monitor for updating the mapping. The simplified attack-detection mechanism is shown in Algorithm 3:
**Algorithm 3** Mechanism of Attack Detection.**Require:** *h*: host; *s*: siwtch; ΔT: time interval  1:Collect data *d* related to *h* and *s* within ΔT  2:Caculate RPM,RFE,APF,ADF from *d*  3:**if** Anormaly type is AIM, **then**  4:    Caculate ESIA from *d*  5:    feature = (RPM,RFE,APF,ADF,ESIA)  6:**else**  7:    Caculate PGV from *d*  8:    feature = (RPM,RFE,APF,ADF,PGV)  9:result = Classfier(feature)10:**if** result = **ATTACK**, **then**11:    Report to mitigator module12:Feedback result on minotor module

### 3.4. Mitigator

The mitigator receives notice from the monitor and detector modules. It extracts the compromised host and installs flow rules to the switch, which the host connected to drop the malicious traffic. As described in OpenFlow specification 1.3, if no output action and no group action were specified in an action set of a flow entry, the packet that matched this flow entry would be dropped [11]. Thus, the mitigator can construct a *Flow-Mod* message with no output action and send it to the specified switch to remove the flows.

## 4. Experiment

This section implements DoSGuard on the Floodlight controller and evaluates it in OpenFlow software environments.

### 4.1. Implementation

**Figure 4:**
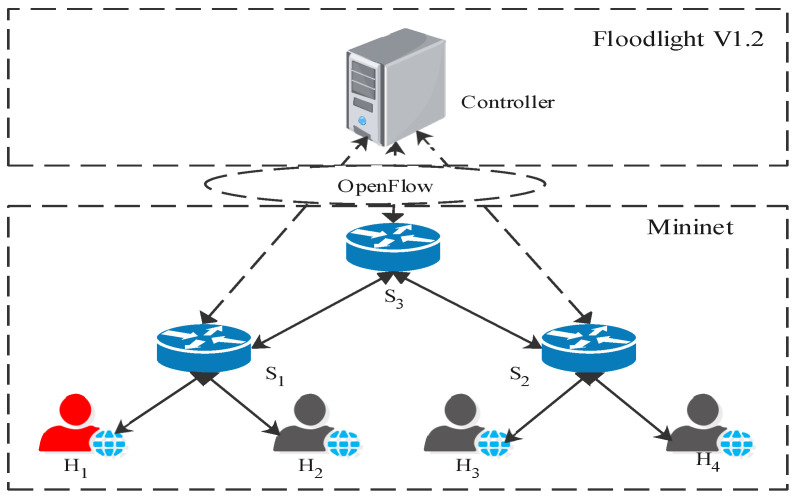
Environment setup.

**Experimental Setup**: We implement the DoSGuard in the Floodlight v1.2 controller and test it under the Mininet environment running on a virtual machine with Ubuntu 16.04 and Intel Core i5-8400 2.80 GHz CPU and 8 GB memory. Figure 4 shows the virtual network topology used for the experiments. We select H1 as the attacker and H2, H3, and H4 as the normal user. The southbound interface is Openflow1.3. We also use Scapy [30], a powerful interactive packet-manipulation program, to generate random packets for launching attacks.

**Dataset and Parameters Setting**: To verify the accuracy of the detection algorithm, we generated a dataset under the topology shown in Figure 4. We use Scapy to generate normal rate packets and attack packets on $H_{1}$, respectively, and send them to other hosts. Through the training sample-generation stage, a total of 25,000 traffic events were collected, including 11,543 normal traffic events and 13,457 attack traffic events. At the same time, we captured 5 h of normal network traffic and counted port changes every 10 s. The maximum port growth rate was 2.7/s. To avoid false alerts, we set δ to 2. In fact, the port growth rate should theoretically be much higher than δ under attack traffic. In practice networks, we can adjust the value according to the network state, referring to the number of hosts in the network and the number of ports used by each host.

### 4.2. Evaluation

We evaluated DoSGuard in terms of effectiveness, advancement, and defense overhead.

#### 4.2.1. Effectiveness Evaluation

To prove that DoSGuard can effectively mitigate DoS attacks against SDN networks, we evaluate the defense effects against the control plane, data plane, and application plane.

**Figure 5:**
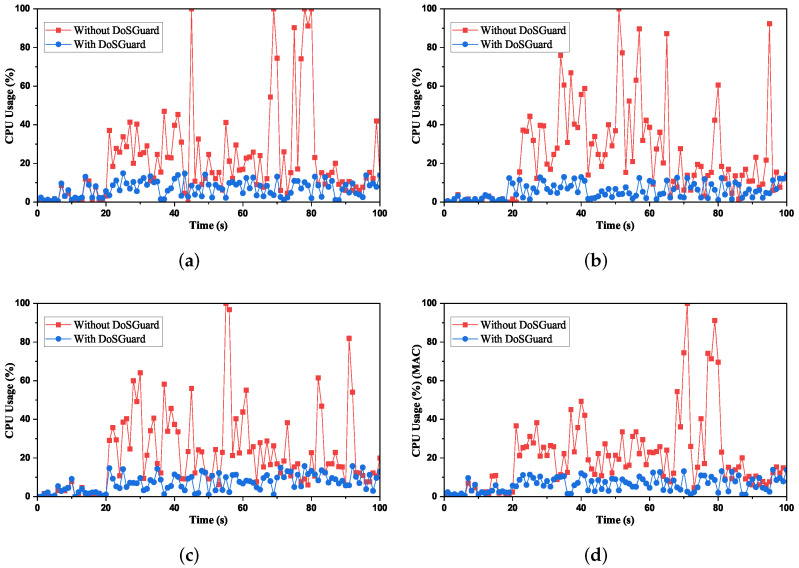
Defense effects on Controller CPU Usage. (**a**) Fake source MAC address. (**b**) Fake source IP address. (**c**) Fake source port number. (**d**) Fake random source address.

**Defense effects on SDN controller**: We measured the controller CPU usage under attack by using the Psutil library [31]. Figure 5 presents the comparison of CPU usage with and without DoSGuard. Figure 5a compares CPU usage under attack by faking the source MAC addresses. As we can see, when the 20-s attack starts without DoSGuard, the controller CPU usage sharply increases because, during the DoS attack, the controller needs to install useless flow rules continuously, and its CPU processing power becomes less available to legitimate flow requests [32]. Since DoSGuard installs rules to drop malicious traffic without being forwarded to the controller, the controller CPU utilization remained at a normal level before and after the attack under the protection of DoSGuard. Figure 5b,c, respectively, compare CPU usage under attack by faking the source IP addresses and ports, which is consistent with the effect in Figure 5a. Figure 5d compares CPU usage under attack by faking random source addresses, including simultaneously spoofing all or any two of them because, in DoSGuard, the anomaly finding is performed sequentially. For example, when a malicious host simultaneously spoofs both MAC and IP address, DoSGuard will judge it as *AIM*. Therefore, the results are no different from others. In summary, all results show that DoSGuard can protect SDN controllers effectively.

**Figure 6:**
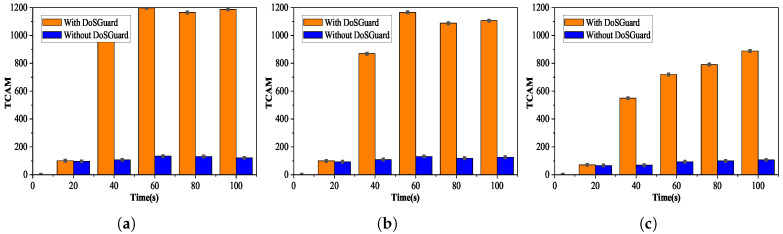
Defense effects on OpenFlow switch. (**a**) Fake source MAC address. (**b**) Fake source IP address. (**c**) Fake source port number.

**Defense effects on OpenFlow switch**: We injected manipulated packets by modifying source addresses (MAC, IP, and port number) and measured the number of flow table entries with and without DoSGuard. The experimental results are depicted in Figure 6. We can see that no matter which attack method is adopted, as shown in Figure 6a–c, when the 20-s attack starts without DoSGuard, the number of flow entries of switch $S_{1}$ increases rapidly, reaching a maximum of 1200 because the controller does not validate the *Packet-In* messages and installs rules for malicious packets. DosGuard can quickly detect attacks and drop malicious traffic, while, with the protection of DoSGuard, the number of flow entries changes steadily without a surge. The results indicate that DoSGuard can protect the flow table resources in OpenFlow switch effectively.

**Figure 7:**
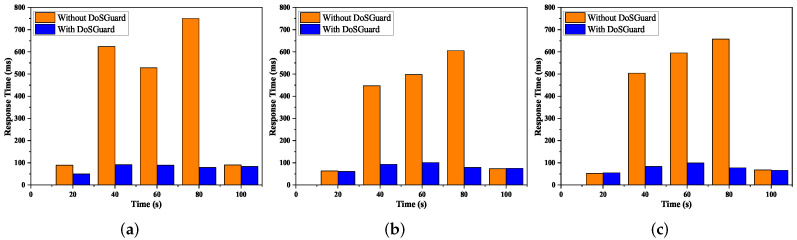
Defense effects on application response time. (**a**) Fake source MAC address. (**b**) Fake source IP address. (**c**) Fake source port number.

**Defense effects on application response time**: We used Floodlight Web GUI to demonstrate the application response time under attack. We continually injected malicious packets and refreshed Web GUI on a timed interval. Figure 7 presents the comparison of application response time with and without DoSGuard. We can see that no matter which attack method is adopted, as shown in Figure 7a–c, after 20 s, when we start the attack without DoSGuard, the application response time increases quickly. The reason is that during the attack, the Floodlight Web GUI does not have enough resources to handle requests. Comparing to the case without the DoSGuard protection, we can see the DoSGuard can effectively defend against the DoS attack, and the application response time is still maintained at an average level.

#### 4.2.2. Advancement Evaluation

First, we proved that SVM is more suitable for our experimental environment and scenarios. Then, we compared DoSGuard with other proposed solutions to indicate that the detection accuracy is better than the state-of-the-art methods.

**Accuracy of detection algorithm**: We evaluated the applicability and efficiency of different machine-learning algorithms in this paper’s scenarios and data. Based on the features mentioned in Section 3.3, we tested the standard classification and clustering algorithms used in anomaly detection, including IsolationForest, SVM, Ran-DomForest, DecisionTree Kmeans, and BayesNet. As a comparison, we use F1 score, precision, and recall to evaluate the performance of different algorithms and schemes. As shown in Formulas (8)–(10), precision represents the accuracy of the detector in part of the data. Recall shows the sensitivity of the detector. F1 score represents the combination of precision and recall. In Formulas (8) and (9),

(1) **TP**: The number of true positives means the detector’s classification is correct and an attack occurs;

(2) **FP**: The number of false positives means the detector’s classification is incorrect and no attack occurs

(3) **FN**: The number of false negatives means the detector’s classification is incorrect and an attack occurs.

(8) $$Precision=\frac{TP}{TP+FP}$$

(9) $$Recall=\frac{TP}{TP+FN}$$

(10) $$F1\;score=2\cdot \frac{Recall\cdot Precision}{Recall+Precision}$$

The results are shown in Table 3. As we can see, the SVM algorithm is superior to others in terms of precision, recall, and F1 score for this paper’s data and features.

Next, we compared with other proposed solutions in the same environment, including the entropy-based detection method [24] and the DoSDefender. The results are shown in Table 4. As we can see, the attack-detection effect of DoSGuard is better than others. The accuracy of both the entropy-based detection method and the DoSDefender depends on the threshold setting, and the false positive is relatively high. After DoSGuard identifies abnormal hosts by verifying information consistency, it detects attacks based on flow and message features, which effectively improves detection accuracy.

#### 4.2.3. Defense Overhead

**Figure 8:**
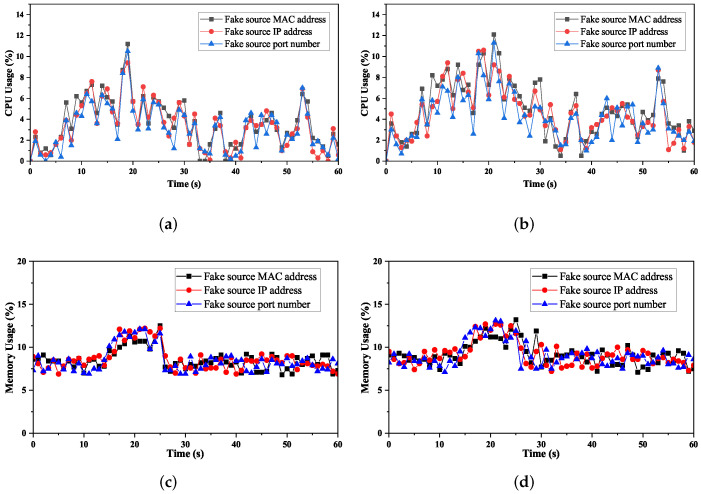
Overhead of the controller under the protection of DoSGuard. (**a**) 1000 packets/s. (**b**) 2000 packets/s. (**c**) 1000 packets/s. (**d**) 2000 packets/s.

We use CPU and memory utilization of the controller to indicate the overhead of the system under different attack rates (e.g., 1000 packets/s, 2000 packets/s). The test time was 60 s, and the attack starts in the 15th second. The evaluation result is presented in Figure 8. As Figure 8a shows, the CPU utilization changes through three stages. Before the attack, the monitor module is active and maintains the mapping relationship between switches and hosts. This process mainly performs packet querying and matching and does not consume many resources, with an average CPU consumption of less than 5%. After the attack starts, the detector collects data, extracts features, and performs attack detection. As a result, the CPU consumption begins to increase gradually, peaking at 11.2%. After completing detection, the mitigator module installs flow rules to drop malicious traffic. Thus, CPU consumption decreased and leveled off, staying around 5%. Therefore, regardless of the attack method, the controller’s average CPU utilization is around 8% with DoSGuard, with acceptable overhead. Figure 8c demonstrates that memory utilization is relatively low and has no considerable fluctuation. By comparing Figure 8a,c and Figure 8c,d, we can see that, although the attack rate increases from 1000 packets/s to 2000 packets/s, both memory and CPU remain at the average level and have no significant change. The results indicate that DoSGuard is robust and stable wand is less affected by the attack power.

**Figure 9:**
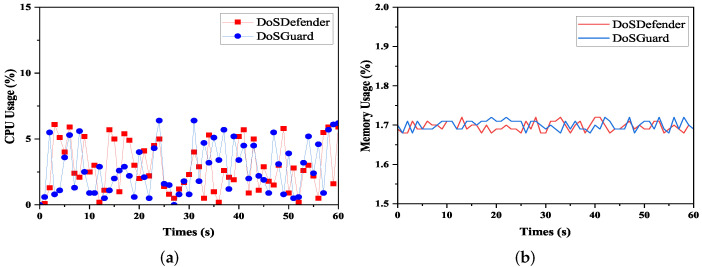
Overload comparison. (**a**) CPU Usage. (**b**) Memory Usage.

We also compared the overhead between DoSGuard and DoSDefender under the normal network. The evaluation result is presented in Figure 9. As we can see, DoSGuard does not incur more overhead than DoSDefender in either CPU utilization or memory utilization. There are two reasons for this. On the one hand, only the monitor is active when no anomaly occurs, and the detector and mitigator are idle. On the other hand, even if an anomaly occurs, although the detector module will perform attack detection, as we have pre-trained the attack detection model, no training process is required after deployment.

From the above results, we can conclude that, no matter the attack method, DoSGuard can ensure better protection to the controller and switches in the network with a limited overhead.

## 5. Advantages and Limitations

This section discusses the advantages and limitations of DoSGuard.

### 5.1. Advantages

The proposed system has the following advantages:

(1) No additional hardware equipment is required: DoSGuard is an extending module of the SDN controller to prevent SDN-aimed DoS attacks. It stands between the controller platform and other controller apps, and all designs conform to the OpenFlow policy. Unlike other approaches, such as [7,9], etc., it does not require additional hardware equipment or any data-plane modifications;

(2) Fast anomaly detection: The monitor constructs the mapping relationship between the switches and the hosts. It realizes the rapid discovery of network anomalies by employing statistical models;

(3) Effective and low overhead: Unlike other DoS detection countermeasures, the monitor wakes up the detector for attack detection only after finding an anomaly, significantly reducing the system’s overhead. At the same time, considering the characteristics of the spoofing source address attack, the detector extracts the most representative features to improve the accuracy of the detection algorithm effectively.

### 5.2. Limitations

The proposed system also has some limitations, which are briefly discussed as follows:

(1) Does not block specific flows: For DoSGuard, once the attack is confirmed, the mitigator will install flow rules to block the compromised host rather than block malicious traffic;

(2) Does not detect attacks for application plane: DoSGuard was mainly designed to protect the control plane and the data plane, for example, the controller and flow tables. It does not defend against threats on the application plane and northbound interfaces (i.e., RESTful API), such as malicious applications or data leakage.

## 6. Conclusions

This paper focuses on the detection and defense methods of DoS attacks carried out by forging source address information in SDN. We propose and implement DoSGuard, a scalable and protocol-independent defense system. As a lightweight extension module on SDN controllers, DoSGuard maintains the information between the switches and the hosts for anomaly detection, utilizing the OpenFlow message and flow features for attack detection. To mitigate attacks, DoSGuard filters malicious packets by installing flow rules. The evaluation results demonstrate the effectiveness of DoSGuard and show that it can prevent SDN-aimed DoS attacks with limited overhead. 

## Figures and Tables

**Table 1:** Comparison with different approaches.

Approach	RAH	RMD	ADC	NAM	HFP	LG
DoSGuard	No	No	No	No	No	No
DosDefender [6]	No	No	No	No	Yes	No
SLUM [7]	No	Yes	Yes	No	No	No
PacketChecker [8]	No	No	No	No	Yes	Yes
INSPECTOR [9]	Yes	No	Yes	No	No	Yes
MBCE&G [10]	No	No	No	Yes	No	No

**RAH** represents the required additional hardware, **RMD** represents the required modifying data plane, **ADC** represents the additional deployment costs, **NAM** represents no attack mitigation, **HFP** represents high falsepositives, and **LG** represents low generality.

**Table 2:** Notations.

Notations	Definition
*S*	Switches in the network
$s_{j}$	Switch *j*
*H*	Hosts in the network
$h_{i}$	Host *i*
*t*	Time interval
δ	Threshold of port growth rate
*T*	Mapping table of switches and hosts
$X_{j}$	Whether *T* contains an entry related to $s_{j}$

**Table 3:** Results of different detection algorithms.

Algorithm	Precision	Recall	F1 Score
SVM	96.77%	100%	98.34%
KMeans	93.33%	93.33%	93.33%
BayesNet	96.55%	93%	94.92%
DecisionTree	96.67%	96.67%	96.67%
RandomForest	93.75%	100%	96.77%
IsolationForest	85.29%	97%	90.62%

**Table 4:** Results of different detection method.

Solution	Precision	Recall	F1 Score
Entropy-based	95.92%	96.25%	96.08%
DoSDefender	97.01%	98.35%	97.68%
DoSGuard	98.72%	98.54%	98.63%

## Data Availability

Not applicable.
